# The influence of chronic diseases and poor working conditions in working life expectancy across educational levels among older employees in The Netherlands

**DOI:** 10.5271/sjweh.4028

**Published:** 2022-06-30

**Authors:** Jolinda LD Schram, Merel Schuring, Karen M Oude Hengel, Alex Burdorf, Suzan JW Robroek

**Affiliations:** 1 Erasmus University Medical Center, Department of Public Health, CA Rotterdam, The Netherlands; 2 Netherlands Organisation for Applied Scientific Research, TNO Work Health and Technology Leiden, The Netherlands

**Keywords:** autonomy, disability benefit, emotional demand, early retirement, physical workload, psychological job demand, social support, socioeconomic inequality, unemployment, working years lost

## Abstract

**Objectives:**

This study aims to estimate the influence of chronic diseases and poor working conditions – across educational levels – on working life expectancy (WLE) and working years lost (WYL) in the Dutch workforce after age 50.

**Methods:**

Information on demographics, chronic diseases, and working conditions from 11 800 Dutch workers aged 50–66 years participating in the Study on Transitions in Employment, Ability and Motivation (STREAM) from 2010/2015 was enriched with monthly information on employment status from Statistics Netherlands up to 2018. In a multistate model, transitions were calculated between paid employment and involuntary exit (disability benefits, unemployment) and voluntary exit (economic inactivity, early retirement) to estimate the impact of education, chronic diseases, and working conditions on WLE and WYL between age 50 and 66.

**Results:**

Workers with a chronic disease (up to 1.01 years) or unfavorable working conditions (up to 0.63 years) had more WYL due to involuntary pathways than workers with no chronic disease or favorable working conditions. The differences in WYL between workers with and without a chronic disease were slightly higher among workers with a lower education level (male: 0.85, female: 1.01 years) compared to workers with a high educational level (male: 0.72, female: 0.82 years). Given the higher prevalence of chronic diseases and unfavorable working conditions, WYL among lower educated workers were higher than among higher educated workers.

**Conclusions:**

The presence of a chronic disease or unfavorable working conditions, more prevalent among lower educated workers, contribute substantially to WYL among older workers. This will increase educational inequalities in working careers.

Policy changes have successfully stimulated older workers to participate in paid employment until a higher age. In The Netherlands, the average retirement age increased from 61 years at the beginning of this century to 65 years and 6 months in 2020 (1). The working life expectancy (WLE) – the time persons are expected to work until retirement – also increased in The Netherlands from an estimated 5.2 years at age 55 in 1992–1996 up to 6.8 years in 2012–2016 (2). WLE differs across educational groups: workers with a low educational level or low occupational class have a lower WLE than workers with a higher socioeconomic position (3–10).

Health and adverse working conditions are important contributors to an early involuntary exit out of paid employment. An international comparative cohort study in 25 European countries showed that the higher prevalence of poor health among lower-educated persons contributed substantially to their higher risk of leaving paid employment due to disability benefits and unemployment (11). With regard to working conditions, the higher prevalence of unfavorable working conditions among low educated workers is partly responsible for their larger displacement from the labor market, especially through involuntary routes (12). Although the role of working conditions and chronic diseases in educational inequalities in paid employment have been well-investigated in recent years (12–15), little is known about the relative importance of chronic diseases and working conditions for educational inequalities in exit from paid employment during the working life course.

In the past few years, several studies have estimated the role of health problems and unfavorable working conditions on WLE. In The Netherlands, 55-year-old workers who rate their health as poor lost up to 0.8 working years before the age of 65 compared to those with a good health (2). A comparable study in Denmark, which also combined survey and register information, reported up to 1.4 years lower WLE at age 55 compared to workers who rate their health as good (16). A register-based study in Denmark reported that high physical work demands reduced WLE until retirement at age 65 by up to 1.2 years for women at age 50 and 1.0 years for men at age 50 (17). The current study contributes to the existing literature by reporting the impact of chronic diseases and unfavorable working conditions on WLE and working years lost (WYL) across educational levels, distinguishing between involuntary pathways (disability benefit and unemployment), voluntary pathways (early retirement and economic inactivity) and mortality. Using a multistate model, this study estimates, across educational levels, the influence of chronic diseases and physical and psychosocial working conditions on WLE and WYL among Dutch workers after age 50.

## Method

### Study design and study population

This longitudinal study was embedded in the Dutch longitudinal Study on Transitions in Employment, Ability and Motivation (STREAM) from 2010 onwards. In STREAM, participants aged ≥45 years were invited to complete an online questionnaire annually in the years 2010–2019, except in 2014 and 2018. In 2010, 15 118 respondents participated and, in 2015, an additional sample of 6738 new participants was added to STREAM. For the current study, only the baseline information of STREAM – from both the 2010 and the 2015 additional sample – was used. The study population of STREAM has been extensively described elsewhere (18). The Medical Ethical Committee of the VU University Medical Centre Amsterdam declared that the Medical Research Involving Human Subjects Act does not apply to STREAM.

STREAM data were enriched by Statistics Netherlands with information on the main income components, social benefit pensions and gross wages, derived from the Dutch tax registers and stored in the social statistical database (SSB) as well as linked to the mortality registries (19). For the current study, STREAM baseline data from 2010 and 2015 were matched with monthly information from SSB (November 2010 until December 2018).

Of the 21 856 STREAM participants, 18 896 gave permission by explicit informed consent to link the questionnaire data to register data of Statistics Netherlands. In total, information of 16 932 respondents could be linked to the register data. For the current study, respondents were included when they were aged 50–66 years (in 2010 or 2015) or turned 50 years during the follow-up period (N=14 939), excluding persons who were self-employed. Of these persons, respondents with information on at least one working condition were included (N=12 957). Finally, respondents were included in the analyses if they either had one of six major chronic diseases or did not have any of the reported diseases at baseline (N=11 800).

### Chronic disease

The presence of a chronic disease was assessed with the following question, “Do you (currently) have one or more of the following chronic diseases, disorders or handicaps?”. Fifteen answer options were provided of which six major chronic diseases were defined by their prevalence: diabetes mellitus, cardiovascular, digestive, psychological, musculoskeletal, and respiratory diseases. Persons with migraine, skin problems, hearing problems, eye problems, epilepsy and life-threatening or other unknown diseases were excluded from the analysis following previous studies (15, 20).

### Education

Education was measured with a question on the highest level of education completed, and categorized into low (primary school, lower and intermediate secondary school, or lower vocational training), intermediate (higher secondary school, or intermediate vocational training), and high (higher vocational education, or university education).

### Working conditions

Physical workload, psychological job demands, autonomy, emotional demands, and social support at work were assessed at baseline. Physical workload included five items on force exertion, static load (standing, posture and kneeling) and vibration (21). Psychological job demands were assessed with four Job Content Questionnaire (JCQ) items on how fast, how much, how hard and how hectic an individual’s work is (22). Autonomy was measured with five items derived from the JCQ about making decisions, deciding the order and speed of conducting tasks, having to find solutions, and being able to take time off (22). Emotional demands were assessed with three items from the Copenhagen Psychosocial Questionnaire (COPSOQ) about emotionally difficult situations, emotional demands, and emotional involvement at work (23). Social support at work was assessed with four items from the COPSOQ concerning how often colleagues and supervisor are willing to help, support and listen to work-related problems (23). All items had the same 5-point answer scale ranging from ‘always’ to ‘(almost) never’. For each working condition a sum score was calculated. The answer categories of the working conditions were recoded in such a way that a higher sum score reflected a higher exposure to poor working conditions. The top quartile of the sum score was used to define a poor working condition, compared to the lower 75% percentile.

### Labor market states

Based on the social statistical database and the mortality statistics, four mutually exclusive states were distinguished: (i) paid employment, (ii) involuntary work exit (unemployment, disability benefits), (iii) voluntary work exit (economic inactivity or (early) retirement), and (iv) death. The first three states are based on the main source of income. Paid employment was defined by main source of income through paid employment, defined by monthly tax transactions. Persons were censored at death.

### Statistical analyses

*Multistate model*. First, based on the information on monthly transitions between states, transition rates were assessed in order to calculate the WLE and WYL. Individuals could move between states over time, allowing persons to move in and out of paid employment. The multi-state model was composed of the previously mentioned four states. A total of nine possible transitions remained, for which a transition matrix was constructed. Calculations were censored at 66 years, and the estimated WLE and WYL is thus based on the transitions from age 50–66 years.

The R package mstate, developed by Putter (24), was used to estimate cumulative transition rates and transition probabilities to fit the multistate models. For each of the nine transitions, gender, educational level, the presence of chronic diseases and exposure to unfavorable working conditions were defined as covariates (25). Using age as the time variable, a Cox proportional hazard model was fitted to estimate the transition rates between states. We assumed the transition rates were only dependent on the current state (Markov assumption). The baseline transition hazards were used to calculate transition probabilities for each of the possible transitions in the model.

*WLE and WYL*. The estimated transition probabilities in the multi-state model were used to calculate the expected length of stay (ELOS) in a specific state, given the current state (ELOS function in the mstate R package). WLE was defined as the number of years in the work state, conditional on being in paid employment at the starting age. Bootstrapping was used to calculate the uncertainty around the ELOS. Bootstrapping consisted of resampling from the study population with replacement. The ELOS was calculated on the bootstrapped population, this was repeated 1000 times. The lower and upper bound of the ELOS were estimated as the 2.5^th^ and 97.5^th^ percentile of the bootstrapped ELOS. Stratified analyses were performed by gender, educational level, and the presence of a chronic disease and by educational level and working conditions. The analyses for table 3 were adjusted for gender. The total WYL due to the non-employment states were calculated as the difference between the age of 66 years and the WLE at age 50.

**Table 1:** Prevalence of gender and unfavorable working conditions stratified by the presence of a chronic disease across educational groups (N=11 800).

	Low education (N=3156)		Intermediate education (N=4594)		High education (N=4050)	
		
	Chronic disease (N=1842)	No chronic disease (N=1314)	Chronic disease (N=2498)	No chronic disease (N=2096)	Chronic disease (N=2003)	No chronic disease (N=2047)

	%	%	%	%	%	%
			
Women	48	46	48	41	44	35
Working conditions						
High physical workload	42	37	30	23	12	7
High job demands	25	21	27	23	35	28
Low autonomy	34	31	31	26	29	22
High emotional demands	14	9	19	13	29	21
Low social support	34	28	27	25	27	24

**Table 2:** Working life expectancy (WLE) and working years lost (WYL) by educational level. [CI=confidence interval.]

	WLE	Involuntary WYL (95% CI)	Voluntary WYL (95% CI)	Mortality WYL (95% CI)
Male				
Low	12.76 (12.25-13.13)	1.48 (1.16-1.79)	1.04 (0.84-1.21)	0.73 (0.44-1.33)
Mid	13.00 (12.65-13.30)	1.31 (1.08-1.54)	1.09 (0.93-1.23)	0.60 (0.39-1.01)
High	13.20 (12.93-13.43)	1.15 (0.98-1.35)	1.14 (1.00-1.27)	0.50 (0.36-0.76)
Female				
Low	12.31 (11.99-12.61)	1.76 (1.51-2.03)	1.40 (1.25-1.55)	0.53 (0.36-0.78)
Mid	12.58 (12.39-12.77)	1.53 (1.40-1.67)	1.45 (1.35-1.54)	0.44 (0.34-0.55)
Low	12.80 (12.68-12.93)	1.33 (1.23-1.42)	1.49 (1.42-1.56)	0.38 (0.31-0.44)

**Table 3:** Working years lost (WYL) due to involuntary (disability benefits and unemployment) and voluntary (economic inactivity and early retirement) exit from paid employment and mortality stratified by gender, educational level, and chronic disease. [CI=confidence interval.]

Educational level	Chronic disease	Involuntary WYL (95% CI)	Voluntary WYL (95% CI)	Mortality WYL (95% CI)
Men				
Low	No chronic disease	1.05 (0.81–1.27)	1.02 (0.85–1.20)	0.54 (0.33–1.00)
	Chronic disease	1.88 (1.48–2.25)	1.06 (0.85–1.20)	0.87 (0.49–1.65)
Intermediate	No chronic disease	0.92 (0.75–1.09)	1.06 (0.92–1.20)	0.48 (0.32–0.76)
	Chronic disease	1.71 (1.40–1.99)	1.12 (0.95–1.28)	0.72 (0.47–1.20)
High	No chronic disease	0.83 (0.69–0.97)	1.11 (0.98–1.24)	0.42 (0.28–0.62)
	Chronic disease	1.55 (1.30–1.80)	1.17 (1.02–1.31)	0.60 (0.39–0.98)
Women				
Low	No chronic disease	1.16 (0.98–1.37)	1.35 (1.19–1.52)	0.42 (0.28–0.62)
	Chronic disease	2.17 (1.86–2.47)	1.44 (1.27–1.60)	0.60 (0.40–0.90)
Intermediate	No chronic disease	1.03 (0.92–1.15)	1.39 (1.28–1.49)	0.37 (0.27–0.48)
	Chronic disease	1.94 (1.75–2.15)	1.49 (1.38–1.60)	0.51 (0.37–0.66)
High	No chronic disease	0.91 (0.83–1.00)	1.43 (1.34–1.51)	0.32 (0.25–0.40)
	Chronic disease	1.73 (1.61–1.88)	1.54 (1.46–1.63)	0.43 (0.33–0.52

## Results

The presence of chronic diseases was higher among persons with a low (58%) and intermediate educational level (54%) compared to persons with a high educational level (49%). Of the six included chronic diseases, the prevalence was highest for musculoskeletal disorders (36%), followed by cardiovascular diseases (11%), respiratory disorders (9%), diabetes (8%), digestive disorders (7%), and psychological disorders (6%). Table 1 shows that workers with a low educational level were also more likely to have a high physical workload, low autonomy and low social support, while workers with a high educational level reported high psychological job demands and high emotional demands more often. Workers with a chronic disease reported more unfavorable working conditions than workers with no chronic disease. In total, 2.7% of the participants died during the follow-up period. This percentage was highest among workers with a low educational level and a chronic disease (3.7%) and lowest among workers with a high educational level without a chronic disease (2.1%).

**Figure 1:**
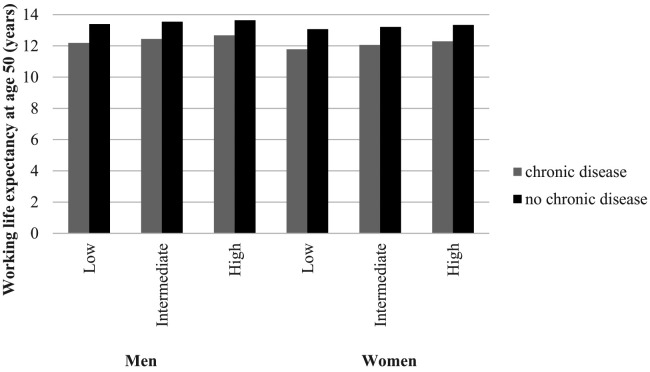
Working life expectancy at age 50 among men and women with (grey) and without (black) a chronic disease stratified by educational level and gender.

Workers with a low educational level have a 0.44 years (men) or 0.49 years (women) lower WLE at age 50 than workers with a high educational level, primarily due to more WYL through involuntary exit from paid employment (table 2). Figure 1 depicts that for all educational levels, workers with a chronic disease had a lower WLE than workers with no chronic disease, ranging from 1.0 year lower WLE among workers with high education to 1.3 years lower for workers with low education. This pattern was similar for men and women.

Most WYL were due to voluntary exit from paid employment, with small educational inequalities and almost no influence of the presence of a chronic disease on voluntary exit (table 3). In contrast, WYL due to involuntary routes were consistently higher among workers with a chronic disease compared to workers without chronic diseases within each educational level (0.72 to 1.01 years). This difference in WYL due to involuntary routes was the highest among workers with a low educational level compared to workers with a high educational level. Workers with a chronic disease also had more WYL due to mortality than workers without a chronic disease with the lowest difference among highly educated women (0.11 years) and the highest difference among lower educated men (0.31 years).

Table 4 shows the influence of various working conditions on WYL across educational levels. Stratification for men and women was not possible due to power considerations. Poor psychosocial work factors were associated with more WYL than physical workload, especially through involuntary exit routes. For involuntary exit from paid employment the largest differences were found for social support at work, with 0.55 years (high education) to 0.63 years (low education) more WYL among persons reporting low social support. Educational inequalities in WYL due to involuntary exit from paid employment varied between 0.03 years for physical workload compared to 0.08 years for social support at work. The influence of unfavorable working conditions on WLE and WYL was quite similar for workers with or without a chronic disease (data not shown).

**Table 4:** Working years lost (WYL) due to involuntary (disability benefits and unemployment) and voluntary (economic inactivity and early retirement) exit from paid employment and mortality stratified by educational level and exposure to unfavourable working conditions.

Educational level	Working condition	Involuntary WYL (95% CI)	Voluntary WYL (95% CI)	Mortality WYL (95% CI)
	**Physical workload**			
Low	Low	1.84 (1.55–2.12)	1.61 (1.44–1.78)	0.44 (0.32–0.66)
	High	1.93 (1.65–2.20)	1.59 (1.41–1.75)	0.48 (0.32–0.71)
Intermediate	Low	1.60 (1.46–1.76)	1.63 (1.52–1.73)	0.38 (0.29–0.47)
	High	1.68 (1.51–1.87)	1.61 (1.48–1.73)	0.41 (0.29–0.53)
High	Low	1.40 (1.31–1.48)	1.64 (1.57–1.71)	0.33 (0.27–0.39)
	High	1.46 (1.31–1.62)	1.63 (1.52–1.75)	0.36 (0.27–0.46)
	**Job demands**			
Low	Low	1.81 (1.56–2.08)	1.63 (1.47–1.79)	0.47 (0.32–0.66)
	High	2.15 (1.83–2.48)	1.56 (1.36–1.75)	0.45 (0.30–0.72)
Intermediate	Low	1.56 (1.42–1.70)	1.65 (1.55–1.74)	0.39 (0.31–0.49)
	High	1.87 (1.66–2.08)	1.58 (1.46–1.72)	0.38 (0.27–0.51)
High	Low	1.34 (1.27–1.43)	1.66 (1.59–1.73)	0.34 (0.28–0.40)
	High	1.61 (1.47–1.77)	1.60 (1.51–1.72)	0.32 (0.24–0.41)
	**Autonomy**			
Low	High	1.80 (1.55–2.08)	1.60 (1.44–1.74)	0.47 (0.33–0.68)
	Low	2.01 (1.72–2.31)	1.64 (1.47–1.83)	0.44 (0.28–0.69)
Intermediate	High	1.58 (1.44–1.71)	1.62 (1.52–1.72)	0.40 (0.31–0.49)
	Low	1.74 (1.55–1.94)	1.65 (1.53–1.78)	0.38 (0.27–0.51)
High	High	1.38 (1.29–1.46)	1.63 (1.57–1.71)	0.34 (0.28–0.40)
	Low	1.51 (1.37–1.66)	1.66 (1.55–1.77)	0.33 (0.25–0.41)
	**Emotional job demands**			
Low	Low	1.88 (1.63–2.15)	1.61 (1.45–1.76)	0.47 (0.31–0.66)
	High	2.11 (1.75–2.50)	1.75 (1.53–1.97)	0.40 (0.23–0.67)
Intermediate	Low	1.62 (1.48–1.75)	1.62 (1.52–1.72)	0.40 (0.32–0.50)
	High	1.82 (1.57–2.08)	1.76 (1.59–1.92)	0.35 (0.22–0.49)
High	Low	1.38 (1.31–1.47)	1.62 (1.55–1.68)	0.35 (0.29–0.40)
	High	1.56 (1.38–1.74)	1.76 (1.63–1.90)	0.30 (0.21–0.41)
	**Social support**			
Low	High	1.66 (1.41–1.91)	1.58 (1.43–1.73)	0.48 (0.34–0.70)
	Low	2.37 (2.04–2.74)	1.70 (1.52–1.87)	0.44 (0.28–0.66)
Intermediate	High	1.46 (1.33–1.59)	1.60 (1.50–1.70)	0.40 (0.31–0.51)
	Low	2.08 (1.86–2.32)	1.72 (1.59–1.85)	0.37 (0.26–0.50)
High	High	1.27 (1.19–1.35)	1.61 (1.55–1.68)	0.34 (0.28–0.40)
	Low	1.82 (1.65–1.97)	1.73 (1.63–1.83)	0.32 (0.24–0.42)

## Discussion

Having a chronic disease (up to 1.01 years) or unfavorable working conditions (up to 0.63 years) are important causes of involuntary working years lost in working careers between age 50 and 66. Across educational levels, differences between these WYL were modest. Hence, lower educated workers will experience more WYL during their working careers, especially due to a higher prevalence of chronic diseases and more unfavorable working conditions. These patterns were similar for men and women.

Previous studies have focused on WLE across educational or occupational groups (3, 5, 6, 8, 9, 26, 27), or on the influence of chronic diseases on WLE (2, 26–28). Their results are in line with the current study, showing the lowest WLE among persons with a low educational level and among workers with a chronic disease. In the current study, the WLE of combined exposure to chronic diseases and a low educational level is explored. This study distinguished WYL due to involuntary exit pathways, defined by unemployment and disability benefits, and voluntary pathways, classified by economic inactivity and early retirement. The results show minor differences between workers with or without chronic disease and with or without unfavorable working conditions in WYL due to *voluntary* exit from paid employment in any of the educational groups. In contrast, the results show clearly that the presence of a chronic disease has a major impact on WLE and WYL due to *involuntary* exit from paid employment across all educational levels. The effect of a chronic disease on WYL was slightly higher among workers with a low compared to high education, respectively 0.09 for men and 0.19 for women. Hence, first and foremost the presence of a chronic disease contributes to WYL, and educational level and working conditions play a substantially lesser role. Given that lower educated workers more often have a chronic disease, as presented in table 1, the proportion of workers with WYL will be higher than among highly educated workers. Therefore, the presence of a chronic disease is an important determinant of educational inequalities in working careers. Although the results are in line with other studies, the differences in WLE across educational levels are lower than in other studies. For example, although Nexø et al (27) also showed a larger influence of a chronic disease than educational level on WLE, the WLE at age 50 differed 1.6 years between those with a low educational level compared to a high educational level. We found a difference of up to 0.5 years. These differences can – at least partly – be explained by different policies and data availability. In The Netherlands, sickness absence is not registered – and persons will typically receive a disability benefit after two years of sickness absence. Technically, workers are regarded as being in paid employment during sickness absence. This can lead to large differences in WLE and WYL in general, and also explain differences across educational groups. Moreover, it needs to be taken into account that the analyses concern persons with paid employment at age 50. Also before this age working years can be lost, and a healthy worker effect can play a role here – which also may underestimate the educational differences in WYL. With regard to working conditions, workers exposed to unfavorable psychosocial working conditions, in particular lack of social support, lose more working years due to involuntary exit from paid employment than workers who are not exposed to these unfavorable psychosocial working conditions. Unfavorable working conditions (eg, low autonomy), except high job demands (eg, high work amount), are more prevalent among workers with a low educational level – and thus also contribute modestly to educational inequalities in working careers. In contrast to psychosocial working conditions, exposure to high physical workload had only a minor influence of 0.1 WYL. One previous study estimated a 1.2 year lower WLE at age 50 among workers with high physical load, and a 0.8 lower WLE among workers with intermediate physical work demands compared to low physical work demands (17). This difference in WLE is much higher than in our study, which can be explained in the light of two main differences between the studies. First, Pedersen (28) included long-term sickness absence as a labor market state. In our study, as mentioned above, sickness absence is part of paid employment, since information is lacking on time spent in full sickness absence or partial return to work. In The Netherlands, a disability benefit is usually granted after a period of two years of sickness absence paid by the employer. Therefore, WYL due to involuntary exit from paid employment will be underestimated in our study. Second, the information in the current study relied on self-reported exposure to working conditions, while the Pedersen study (17) used a job exposure matrix based on specific ergonomic postures. In the latter study three categories of exposure to physical demands were formed, with a prevalence of 3-4% for high work demands – which is much lower than the prevalence up to 42% in our study. It is likely that the high physical work demands in the Pedersen study are more strenuous compared to our study.

Working conditions and chronic diseases are also interrelated. Leijten et al (29) found that favorable working conditions can reduce the risk of exit from paid employment due to disability benefits. According to the capability approach, the ability to perform at work does not only depend on individual characteristics but also on the work context (eg, with an adequate HRM policy) (30). The difference in WYL between persons with and without chronic diseases asks for a more inclusive workforce, in which employers are encouraged to facilitate a healthy work environment with specific attention for workers with a chronic disease. It would be of interest to further unravel the complex interplay between educational level, working conditions and chronic diseases on WLE and WYL.

### Strengths and limitations

The strength of this study is the enrichment of questionnaire data with register data containing objective information on work status. The measures of WLE and WYL are relevant metrics, summarizing information over the working life course. This information can also be used to study the impact of policy changes (31). The measure of WLE may for example guide policymakers in the discussion concerning elevating retirement age. There is, however, no clear guidance as when a WLE or WYL is low or high, and when group differences are calling for action as this will depend highly of the particular context of the situation studied. Another limitation concerns a lack of power to distinguish specific underlying pathways of exit from paid employment or to investigate the influence of specific chronic diseases on WLE and WYL. As also concluded in a recent narrative review (32), it would be of interest to further disentangle unemployment and disability benefits as to their contribution to the differences in WYL for workers with chronic diseases or workers exposed to unfavorable working conditions. In addition, it would be of interest to take into account the accumulation of exposures to working conditions and chronic diseases over time. Another limitation concerns that transitions to death were uncommon and therefore provide less reliable results.

### Concluding remarks

This study showed that workers with a chronic disease or unfavorable working conditions lose more working years due to involuntary exit from paid employment than workers with no chronic disease or with favorable working conditions. Workers with a low educational level lose more working years due to the presence of a chronic disease and unfavorable psychosocial working conditions than workers with a higher educational level. This will increase educational inequalities in working careers.
